# Radiotherapy with 5 × 5 Gy for Personalized Treatment of Malignant Epidural Compression of the Myelon: Long-Term Results of the PRE-MODE Trial

**DOI:** 10.3390/jpm15120577

**Published:** 2025-11-28

**Authors:** Dirk Rades, Darejan Lomidze, Carlos Ferrer-Albiach, Antonio J. Conde-Moreno, Barbara Segedin, Blaz Groselj, Raquel Ciervide Jurio, Jon Cacicedo

**Affiliations:** 1 Department of Radiation Oncology, University Medical Center Schleswig-Holstein, Lübeck Campus, 23538 Lübeck, Germany; 2 Department of Radiation Oncology, University of Lübeck, 23562 Lübeck, Germany; 3 Radiation Oncology Department, Tbilisi State Medical University, 0177 Tbilisi, Georgia; dlomidze@hotmail.com; 4 Radiation Oncology Department, Ingorokva High Medical Technology University Clinic, 0177 Tbilisi, Georgia; 5 Department of Radiation Oncology, Consorcio Hospital Provincial de Castellon, 12002 Castellon, Spain; carlos.ferrer@hospitalprovincial.es (C.F.-A.); conde_ant@gva.es (A.J.C.-M.); 6 Department of Radiation Oncology, University and Polytechnic Hospital La Fe, 46026 Valencia, Spain; 7 Department of Radiotherapy, Institute of Oncology Ljubljana, 1000 Ljubljana, Slovenia; bsegedin@onko-i.si (B.S.); bgroselj@onko-i.si (B.G.); 8 Department of Radiotherapy, Faculty of Medicine, University of Ljubljana, 1000 Ljubljana, Slovenia; 9 Department of Radiation Oncology, University Hospital HM Sanchinarro, 28050 Madrid, Spain; rciervide@hmhospitales.com; 10 Radiation Oncology Department, Hospital Universitario Cruces, 48903 Barakaldo, Spain; jon.cacicedofernandezbobadilla@osakidetza.eus; 11 Radiation Oncology Department, Biobizkaia Health Research Institute, 48903 Barakaldo, Spain

**Keywords:** malignant epidural compression of the myelon, radiotherapy, clinical trial, local progression-free survival, long-term-results

## Abstract

Background/Objectives: Despite increasing use of upfront decompressive surgery for malignant epidural compression of the myelon (MESCC), a substantial number of affected patients still receive radiotherapy (RT) alone. Many of these patients would benefit from a personalized treatment approach including the most appropriate dose-fractionation regimen. The PRE-MODE trial (NCT03070431) compared precision RT with 5 × 5 Gy (prospective cohort, *n* = 40) to conventional RT with 5 × 4 Gy (historical control, *n* = 676)). After propensity-score matching, 5 × 5 Gy resulted in significantly increased local progression-free survival (LPFS) at 6 months than 5 × 4 Gy. The question arose whether this benefit is still present after a longer period of follow-up. Methods: For this additional study, supplementary data were retrospectively captured, resulting in prolongation of follow-up until 24 months. Results: 5 × 5 Gy resulted in LPFS of 80.9% at each investigated time point (12, 18, and 24 months) without reported radiation myelopathy. Moreover, 5 × 5 Gy showed a trend towards improved LPFS after 12 (*p* = 0.070), 18 (*p* = 0.060), and 24 (*p* = 0.054) months. Similarly to the original PRE-MODE trial, OS-rates were not significantly different in the dose groups of this supplementary study. Conclusion: Since 5 × 5 Gy resulted in excellent long-term LPFS and showed a trend towards better outcomes up to 24 months following RT, it appears preferable to 5 × 4 Gy and will contribute to the personalized treatment of patients with MESCC who are assigned to RT alone without upfront neurosurgical intervention.

## 1. Introduction

Malignant epidural compression to the myelon (MESCC) generally needs immediate intervention and may be found in up to every tenth patient with a known malignancy [1,2]. For several decades, radiotherapy (RT) alone was the standard treatment for this situation. A randomized trial revealed a benefit for preceding decompressive surgery in selected patients [3]. Since then, surgical interventions have become increasingly popular for MESCC. A considerable number of patients, especially patients with a limited performances status, still receive radio-therapy (RT) alone [1]. Many of these patients will benefit from a personalized treatment approach including the most appropriate dose-fractionation regimen. Patients with MESCC assigned to RT alone often receive 5 × 4 Gy over one week to avoid unnecessary treatment sessions for these patients generally presenting with pain and immobility [1,2]. However, a previous prospective study showed that longer-course programs including 30 Gy over two weeks resulted in better local progression-free survival (LPFS) than 5 × 4 Gy or 1 × 8 Gy [4]. As in cases of local failure, re-treatment to improve neurologic deficits and pain may be difficult, and long-term LPFS is an important treatment goal. The prospective PRE-MODE trial (NCT03070431) addressed both aspects, a shorter overall treatment time and favorable LPFS [5]. It compared RT with 5 × 5 Gy (prospective cohort) to 5 × 4 Gy (historical control). To kill tumor cells, the equivalent dose in 2-Gy fractions (EQD2) of 5 × 5 Gy is almost the same as of 10 × 3 Gy [6]. 5 × 5 Gy was regarded safe, because its EQD2 is less than the tolerance dose of the myelon of 45 Gy. Radiation myelopathy was mainly observed only after an EQD2 higher than 50 Gy [7,8].

After propensity-score matching in the PRE-MODE trial, 5 × 5 Gy resulted in longer LPFS than 5 × 4 Gy [5]. The follow-up was limited to 6 months in the prospective cohort and censored at 6 months in the historical control group. The question arose whether the improvement in LPFS does persist longer than 6 months. Therefore, this secondary study was performed. The contributing centers collected additional data of the patients of the PRE-MODE trial still alive after 6 months. The follow-up in the control group was not limited to 6 months as in the original PRE-MODE trial. The additional data enabled us to compare 5 × 5 Gy and 5 × 4 Gy up to 24 months following RT. If the superiority of 5 × 5 Gy will last longer than 6 months, this less commonly used regimen will be an additional option to improve the personalized treatment of patients with MESCC treated with RT alone.

## 2. Materials and Methods

Forty patients of the PRE-MODE trial (clinicaltrials.gov, identifier NCT0307043, first posted on 03-MAR-2017) received 5 × 5 Gy of volumetric modulated arc therapy (*n* = 38) or fixed-field intensity-modulated RT (*n* = 2) for weakness of the legs as a consequence of MESCC, which was confirmed by magnetic resonance imaging or computed tomography. Thirty-nine patients were treated at university hospitals, and one patient at a provincial hospital. The dose at the myelon was required to be 101.5% or less of the prescribed dose, meaning a myelopathy risk of less than 0.03% [5]. According to the study protocol, the planned target volume was required to encompass the vertebrae affected by MESCC and a cranio-caudal margin of 10 mm and to be covered by the 95% isodose line. The maximum EQD2 of 43.8 Gy was also significantly lower than the tolerance dose of bone [9]. The EQD2 for the esophagus, the heart, and the lungs were required to be less than 34 Gy, less than 26 Gy, and less or equal to 7 Gy, respectively [9].

Patients with previous RT or surgery in the spinal parts currently affected by MESCC, involvement of cervical spinal cord only, motor deficits due to other causes, or a clear indication for upfront neurosurgery were not included [5]. Investigated endpoints included LPFS up to 6 months following RT (primary endpoint), impact of RT on neurologic and gait functions, pain, distress, and overall survival (OS). PRE-MODE patients were compared to members of the historical control group who received 5 × 4 Gy of conventional RT and met the same eligibility criteria as the PRE-MODE cohort. For better comparability, follow-up in the control group was censored at 6 months.

For comparisons between the PRE-MODE cohort and the control group, propensity-score methods were applied to consider differences in baseline characteristics, and reduce the risk of biases [10]. For propensity scores, ten covariates were considered. These included age (≤65 years vs. ≥66 years), gender, tumor type (breast cancer vs. prostate cancer vs. myeloma/lymphoma vs. lung cancer vs. other malignancies), time from tumor diagnosis to MESCC (≤15 vs. >15 months), affected vertebrae (*n* = 1 − 2 vs. *n* ≥ 3), other bone lesions (no vs. yes), visceral lesions (no vs. yes), dynamic/time of the development of motor weakness (1 − 7 vs. 8 − 14 vs. >14 days), gait function (not ambulatory vs. ambulatory with or without aid), and performance score (1 − 2 vs. 3 − 4). The distribution of these covariates was previously reported [5]. Subsequently, propensity scores were used to assign the patients to five strata. Cochran–Mantel–Haenszel tests were applied for evaluation of the removal of biases. Statistical analyses were performed with SAS software (version 9.4; SAS, Cary, NC, USA). Further details of materials and methods including statistical approaches and sample size calculations were presented when reporting the PRE-MODE trial [5]. The TREND Statement Checklist can be found in Supplementary File S1.

In the original PRE-MODE trial, 40 patients of the prospective cohort and 664 of 676 subjects of the historical control group did qualify for the propensity-score analyses [5]. For this supplementary retrospective study, the same patients were used. For 16 patients of the PRE-MODE cohort and 259 patients of the control group, follow-up was extended. The additional data for the PRE-MODE cohort were collected between 03 September 2025 and 25 September 2025. The current study was approved by the local Ethics Committee in Lübeck, Germany (2025-403); so was the original PRE-MODE trial (16-163) [5]. Since the data of some endpoints would not change after the evaluation at 6 months (impact of RT on motor and sensory functions, post-RT gait function, sphincter dysfunction) or were not available (relief of pain and distress), the present study focused on LPFS and OS. The main goal was to investigate whether the superiority of 5 × 5 Gy with respect to LPFS was present at 12, 18, and 24 months after irradiation. LPFS was defined as absence of progression of motor weakness during the RT course and absence of a recurrence of MESCC associated with motor weakness after completion of the radiation treatment. During the follow-up period that surpassed 6 months, patients did not undergo a structured diagnostic program but were seen and received a clinical examination and diagnostic imaging only in cases of recurrent neurologic symptoms. For the analyses of LPFS and OS, the same statistical methods including the propensity-score matching were applied as in the original PRE-MODE trial [5].

## 3. Results

In the PRE-MODE cohort, the LPFS rates after 12, 18, and 24 months were always 80.9% (descriptive analyses), and the OS rates were 32.9%, 29.9%, and 26.6%, respectively. Radiation myelopathy, vertebral fractures, or other potential Late toxicities were not reported in the PRE-MODE cohort. In the historical control group, the LPFS rates after 12, 18, and 24 months were 68.6%, 65.0%, and 58.7% (descriptive analyses), and the OS rates were 35.4%, 29.2%, and 23.0%, respectively. After propensity-score matching, 5 × 5 Gy (PRE-MODE cohort) showed a trend towards improved LPFS after 12 (*p* = 0.070), 18 (*p* = 0.060), and 24 (*p* = 0.054) months when compared to 5 × 4 Gy (historical control group) (Table 1). The OS rates were not significantly different between both cohorts after 12 (*p* = 0.735), 18 (*p* = 0.737), and 24 (*p* = 0.663) months, respectively (Table 2).

## 4. Discussion

A variety of fractionation-regimens is in use worldwide for patients with MESCC who are candidates for RT alone [1,2]. The decision regarding the preferred individual regimen should be based on the patient’s personal situation and expectations. Moreover, it depends on several aspects including national customs and personal experiences of the treating radiation oncologists. One commonly used multi-fraction regimen is 5 × 4 Gy over one week [1,2,11,12,13,14]. However, a prospective trial of 265 patients with motor deficits caused by MESCC demonstrated that shorter-course regimens with lower total doses, namely 5 × 4 Gy and 1 × 8 Gy, were associated with significantly more in-field recurrences of MESCC after 6 and 12 months when compared to longer-course regimens including 10 × 3 Gy, 15 × 2.5 Gy, and 20 × 2 Gy [4]. In that trial, the most common dose-fractionation regimens were 5 × 4 Gy in 107 patients and 10 × 3 Gy in 111 patients, respectively. Thus, the trial mainly compared these two regimens. The better outcome after longer-course RT was explained by the higher EQD2 of 32.5 Gy in comparison to 23.3 Gy [6]. In order to avoid unnecessary treatment sessions for individual patients, the PRE-MODE trial investigated a less commonly used shorter-course regimen: 5 × 5 Gy over one week with an EQD2 similar to that of 10 × 3 Gy (31.3 Gy) [5]. The main endpoint was LPFS, which was significantly better in the 5 × 5 Gy group (*p* = 0.026). However, the follow-up of the PRE-MODE trial was limited to 6 months. We felt that it would be interesting to know whether the superiority of 5 × 5 Gy over 5 × 4 Gy would still be present after a longer period of follow-up. Therefore, the present secondary study was performed that evaluated LPFS and OS at 12, 18, and 24 months following RT. The LPFS rates after 5 × 5 Gy were high, namely 80.9% at all three time points. The comparability of the long-term results of LPFS with the findings of previous studies using RT alone appears limited, because only very few studies reported LPFS rates beyond 6 months. A retrospective matched-pair study compared 10 × 3 Gy (N = 191) to 15 × 2.5 Gy or 20 × 2 Gy (N = 191) in patients with MESCC and a relatively good OS prognosis [15]. After 10 × 3 Gy, the LPFS rates at 12, 18, and 24 months were 84%, 80%, and 68%, respectively. After 15 × 2.5 Gy or 20 × 2 Gy, LPFS rates were 90% at all three time points. Thus, 5 × 5 Gy appears similarly effective as 10 × 3 Gy, which is supported by the almost identical EQD2 for tumor cell kill [6,7]. LPFS after 15 × 2.5 Gy or 20 × 2 Gy in the previous study was better than after 5 × 5 Gy in the current study [15]. However, this comparison is of limited value, because all patients in the previous study had a relatively good OS prognosis [15]. The same limitation holds true for a recent phase 2 trial performed in 50 patients with a relatively good OS prognosis, where the 12-month LPFs was 96.8% after 18 × 2.333 Gy or 15 × 2.633 Gy [16]. In a preliminary report of a prospective non-randomized study comparing 1 × 8 Gy or 5 × 4 Gy to 10 × 3 Gy, 15 × 2.5 Gy or 20 × 2 Gy in 231 patients (regardless of their OS prognosis), 1-year LPFS rates were 72% and 55%, respectively, i.e., worse than after 5 × 5 Gy in this study [17].

Despite the favorable LPFS rates at 12, 18, and 24 months after 5 × 5 Gy in the present study, these rates were not significantly higher when compared to 5 × 4 Gy after propensity-score matching methods were applied. At least a trend was found for the difference in LPFS between both dose groups with *p*-values of 0.070, 0.060, and 0.054 at 12, 18, and 24 months, respectively. When looking at the differences given in percentage (80.9% vs. 68.6%, 65.0%, and 58.7% at 12, 18 and 24 months, respectively), 5 × 5 Gy may be considered superior to 5 × 4 Gy, although statistical significance was narrowly missed. The *p*-values might have been influenced by the relatively small sample size in the 5 × 5 Gy group. The OS rates at 12, 18, and 24 months were similar in both groups, which agrees with the results of the original PRE-MODE trial [5]. The 12-month OS rates in the present study were higher (32.9% after 5 × 5 Gy and 35.4% after 5 × 4 Gy, respectively), than the 12-month OS rates in the final report of the previous prospective study (30% after longer-course and 23% after shorter-course RT) [4]. Since LPFS is an important goal in MESCC treatment, we feel that 5 × 5 Gy should be preferred to 5 × 4 Gy. It appears to be more effective, although statistical significance was not reached, and is not more time consuming for patients and staff members than 5 × 4 Gy. 5 × 5 Gy appears to be a reasonable additional option for the personalized treatment of patients with MESCC assigned to RT alone. This regimen combines a comparably high EQD2 for tumor cells and a low number of RT fractions. Therefore, it may be used for selected patients instead of the more established regimens 5 × 4 Gy and 10 × 3 Gy [1,2]. As longer-course regimens with a higher EQD2 were found to increase LPFS in patients with better OS prognoses, 5 × 5 Gy cannot be recommended for this group and should be considered as a personalized approach for patients with poor or intermediate prognoses [18].

*Limitations:* When following this recommendation, one should understand the limitations of the present study. Despite the use of propensity-score methods, a risk of bias remains due to the retrospective nature of the data of the historical control group and the additional data collected for the PRE-MODE cohort. For example, the superiority of 5 × 5 Gy in this study may have been influenced by institutional or technical differences. Moreover, patients of the historical control group received conventional RT, whereas precision techniques were used for the PRE-MODE cohort. When considering this aspect, it may be questioned whether the observed benefit was attributed more to advanced delivery techniques rather than the dose escalation itself. The sample size in the 5 × 5 Gy group was comparatively small, which led to low numbers of subjects in the subgroups stratified by propensity score quintiles (Table 1 and Table 2). Due to the fact that the patients did not undergo a structured follow-up program later than 6 months following their RT course, in-field recurrences may have been missed. Due to these limitations, an additional study, ideally a randomized trial in a larger patient cohort, that also uses precision RT for the control group is required. Precision RT has already become quite popular for definitive treatment of spinal metastases, spinal re-irradiation, and post-operative treatment following separation surgery for MESCC [18,19,20,21,22,23,24,25,26,27,28,29,30,31,32,33,34]. Furthermore, the findings of the present study may not be generalized to patients with MESCC receiving other treatments than irradiation alone [18,19,20,21,22,23,24,25,26,27,28,29,30,31,32,33,34]. Moreover, as patients with affection of the cervical spine alone were not part of this study, the results may not hold true for this clinical situation.

## 5. Conclusions

RT with 5 × 5 Gy resulted in high LPFS rates after 12, 18, and 24 months and showed a trend towards improved LPFS in comparison to 5 × 4 Gy at each of these time points. Since 5 × 5 Gy appears more effective than 5 × 4 Gy and does not require more treatment sessions, it can be considered preferable to 5 × 4 Gy and will contribute to the personalized treatment of patients with MESCC assigned to RT alone. Because of the limitations of this study, additional data, ideally from a randomized trial, would be necessary to support this recommendation.

## Figures and Tables

**Table 1:** Analysis of local progression-free survival at 12, 18, and 24 months following RT, stratified by propensity score quintiles.

	Quintile 1		Quintile 2		Quintile 3		Quintile 4		Quintile 5	
	Control Group *n* (%)	PRE-MODE *n* (%)	Control Group *n* (%)	PRE-MODE *n* (%)	Control Group *n* (%)	PRE-MODE *n* (%)	Control Group *n* (%)	PRE-MODE *n* (%)	Control Group *n* (%)	PRE-MODE *n* (%)
Local failure up to 12 months following RT										
No	160 (76.6)	6 (75.0)	203 (75.5)	8 (100)	111 (75.5)	8 (88.9)	14 (73.7)	7 (100)	17 (85.0)	7 (87.5)
Yes	49 (23.4)	2 (25.0)	66 (24.5)	0 (0.0)	36 (24.5)	1 (11.1)	5 (26.3)	0 (0.0)	3 (15.0)	1 (12.5)
*p* = 0.070										
Local failure up to 18 months following RT										
No	158 (75.6)	6 (75.0)	203 (75.5)	8 (100)	109 (74.1)	8 (88.9)	14 (73.7)	7 (100)	17 (85.0)	7 (87.5)
Yes	51 (24.4)	2 (25.0)	66 (24.5)	0 (0.0)	38 (25.9)	1 (11.1)	5 (26.3)	0 (0.0)	3 (15.0)	1 (12.5)
*p* = 0.060										
Local failure up to 24 months following RT										
No	158 (75.6)	6 (75.0)	202 (75.1)	8 (100)	107 (72.8)	8 (88.9)	14 (73.7)	7 (100)	17 (85.0)	7 (87.5)
Yes	51 (24.4)	2 (25.0)	67 (24.9)	0 (0.0)	40 (27.2)	1 (11.1)	5 (26.3)	0 (0.0)	3 (15.0)	1 (12.5)
*p* = 0.054										

**Table 2:** Analysis of overall survival at 12, 18, and 24 months following RT, stratified by propensity score quintiles.

	Quintile 1		Quintile 2		Quintile 3		Quintile 4		Quintile 5	
	Control Group *n* (%)	PRE-MODE n (%)	Control Group *n* (%)	PRE-MODE *n* (%)	Control Group *n* (%)	PRE-MODE *n* (%)	Control Group *n* (%)	PRE-MODE *n* (%)	Control Group *n* (%)	PRE-MODE *n* (%)
Death up to 12 months following RT										
No	93 (44.5)	2 (25.0)	81 (30.1)	4 (50.0)	66 (44.9)	3 (33.3)	4 (21.1)	3 (42.9)	9 (45.0)	2 (25.0)
Yes	116 (55.5)	6 (75.0)	188 (69.9)	4 (50.0)	81 (55.1)	6 (66.7)	15 (78.9)	4 (57.1)	11 (55.0)	6 (75.0)
*p* = 0.735										
Death up to 18 months following RT										
No	89 (42.6)	2 (25.0)	72 (26.8)	4 (50.0)	61 (41.5)	2 (22.2)	3 (15.8)	3 (42.9)	9 (45.0)	2 (25.0)
Yes	120 (57.4)	6 (75.0)	197 (73.2)	4 (50.0)	86 (58.5)	7 (77.8)	16 (84.2)	4 (57.1)	11 (55.0)	6 (75.0)
*p* = 0.737										
Death up to 24 months following RT										
No	87 (41.6)	2 (25.0)	69 (25.7)	4 (50.0)	56 (38.1)	2 (22.2)	3 (15.8)	3 (42.9)	9 (45.0)	1 (12.5)
Yes	122 (58.4)	6 (75.0)	200 (74.3)	4 (50.0)	91 (61.9)	7 (77.8)	16 (84.2)	4 (57.1)	11 (55.0)	7 (87.5)
*p* = 0.663										

## Data Availability

Further information regarding the PRE-MODE trial is available at clinicaltrials.gov (identifier: NCT03070431). The original contributions presented in the present study are included in the article. Further inquiries can be directed to the corresponding author.

## Supplementary Materials

The following supporting information can be downloaded at: https://www.mdpi.com/article/10.3390/jpm15120577/s1, File S1: TREND Statement Checklist.

## Abbreviations

The following abbreviations are used in this manuscript:

AUC	Area under the Curve
EQD2	Equivalent Dose in 2-Gy Fractions
LPFS	Local Progression-Free Survival
MESCC	Malignant Epidural Spinal Cord Compression
OS	Overall Survival
ROC	Receiver Operating Characteristic
RT	Radiotherapy
